# GH deficiency status combined with GH receptor polymorphism affects response to GH in children

**DOI:** 10.1530/EJE-15-0474

**Published:** 2015-12

**Authors:** Armand Valsesia, Pierre Chatelain, Adam Stevens, Valentina A Peterkova, Alicia Belgorosky, Mohamad Maghnie, Franco Antoniazzi, Ekaterina Koledova, Jerome Wojcik, Pierre Farmer, Benoit Destenaves, Peter Clayton, the PREDICT Investigator group

**Affiliations:** 1 Merck Serono SA, Geneva, Switzerland; 1 Département de Pédiatrie, Hôpital Mère-Enfant, Université Claude Bernard, Lyon, France; 2 Federal State Institution ‘Endocrinology Scientific Center of Russian Medical Technology’, Moscow, Russia; 3 Endocrine Service, Hospital de Pediatría Garrahan, Ciudad Autónoma de Buenos Aires, Buenos Aires, Argentina; 4 IRCCS Istituto Giannina Gaslini di Genova Clinica Pediatrica, Università di Genova, Genova, Italy; 5 Pediatra d.U. Azienda Ospedaliera, Universitaria Integrata Università di Verona, Verona, Italy; 6 Manchester Academic Health Sciences Centre, Royal Manchester Children's Hospital, 5th Floor, Oxford Road, Manchester, M13 9WL, UK

## Abstract

Meta-analysis has shown a modest improvement in first-year growth response to recombinant human GH (r-hGH) for carriers of the exon 3-deleted GH receptor (GHRd3) polymorphism but with significant interstudy variability. The associations between GHRd3 and growth response to r-hGH over 3 years in relation to severity of GH deficiency (GHD) were investigated in patients from 14 countries. Treatment-naïve pre-pubertal children with GHD were enrolled from the PREDICT studies (NCT00256126 and NCT00699855), categorized by peak GH level (peak GH) during provocation test: ≤4 μg/l (severe GHD; *n*=45) and >4 to <10 μg/l mild GHD; *n*=49) and genotyped for the GHRd3 polymorphism (full length (fl/fl, fl/d3, d3/d3). Gene expression (GE) profiles were characterized at baseline. Changes in growth (height (cm) and SDS) over 3 years were measured. There was a dichotomous influence of GHRd3 polymorphism on response to r-hGH, dependent on peak GH level. GH peak level (higher vs lower) and GHRd3 (fl/fl vs d3 carriers) combined status was associated with height change over 3 years (*P*<0.05). GHRd3 carriers with lower peak GH had lower growth than subjects with fl/fl (median difference after 3 years −3.3 cm; −0.3 SDS). Conversely, GHRd3 carriers with higher peak GH had better growth (+2.7 cm; +0.2 SDS). Similar patterns were observed for GH-dependent biomarkers. GE profiles were significantly different between the groups, indicating that the interaction between GH status and GHRd3 carriage can be identified at a transcriptomic level. This study demonstrates that responses to r-hGH depend on the interaction between GHD severity and GHRd3 carriage.

## Introduction

Response to growth hormone (GH) therapy is variable in subjects who are GH-deficient (1). Factors including age, BMI, and gender have a role in this individual variability, while genetic factors influencing response to GH are actively being investigated. Dos Santos *et al*. (2) showed that the exon 3-deleted GH receptor (GHRd3) was associated with better growth response to recombinant human GH (r-hGH) treatment in children with idiopathic short stature and children born small for gestational age (SGA). This initial observation was confirmed in patients with Turner syndrome (3), GH deficiency (GHD) (4, 5), those born SGA (6), and those with idiopathic short stature (7). However, several independent studies did not detect such associations (8, 9, 10, 11, 12, 13, 14, 15). Confounding factors, such as severity of GHD, may explain conflicting data between studies.

With the aim of clarifying this controversy, we analyzed the clinical and genetic data generated in the PREDICT and the PREDICT long-term follow-up studies (ClinicalTrials.gov Identifiers: NCT00256126 and NCT00699855) (16). In this observational study, 94 subjects with GHD (45 classified as having severe GHD and 49 as having mild GHD) were followed-up during 3 years of r-hGH therapy. Auxological measurements were taken every year, and change in growth relative to baseline was used to measure the effect of the therapy and tested for association with the GHRd3 polymorphism. Changes in serum biomarkers were measured after 1 month of r-hGH therapy and were also tested for association with the GHRd3 polymorphism. In all analyses, the effect of GHRd3 carriage in relation to GHD severity was assessed. In order to identify potential mechanisms at the cellular level associated with this interaction, baseline gene expression (GE) profiles were analyzed.

## Materials and methods

### Study design

The PREDICT Long-Term Follow-up of Predictive Markers study is an open-label, multicenter study in subjects with GHD. Inclusion criteria included: i) subjects who had completed the PREDICT study assessment of serum biomarkers over 1 month of r-hGH treatment) and who had been followed-up for at least 1 year after completion of the PREDICT study, while still on r-hGH therapy and ii) parental or guardian written informed consent, given before any data collection. Subjects on an investigational drug or participating in another interventional clinical trial since completion of the PREDICT trial were not included. The analyses were restricted further to subjects with GHD who had participated in all 3 years of follow-up.

Data on 94 subjects with GHD, from the per-protocol population, were evaluated (see baseline characteristics in Table 1). All patients were prepubertal at enrolment, with 52% remaining prepubertal at year 2 and 41% at year 3. The diagnosis of GHD was based on two different stimulation tests both with a peak GH value <10 μg/l. The stimulation tests and GH assay used were chosen by the local centre. The most common tests used were: Insulin tolerance test, arginine and clonidine. The combined growth hormone-releasing hormone-arginine test was not used. Patients were selected for r-hGH treatment, based on the criteria used in the local units, in order to capture the full range of GHD patients currently being treated with r-hGH. Patients with GHD associated with etiologies such as CNS tumors with or without cranial irradiation were excluded. GHD was further classified by GH peak during GH stimulation testing as severe (GH peak ≤4 μg/l) or mild (GH peak >4 to <10 μg/l), based on the highest peak level in two independent stimulation tests. Serum biomarkers collected at baseline and after 1 month in NCT00256126 were assayed in a central laboratory (qLAB, Livingston, Edinburgh, UK) (17). Serum insulin-like growth factor1 (IGF1) and insulin-like growth factor-binding protein 3 (IGFBP3) levels were measured using chemiluminescent immunoassays (DPC Immulite 2000: Siemens, Healthcare Diagnostics, Norwood, MA, USA). Levels were converted to SDS using relevant reference data (18). Other parameters (thyroxine (T_4_), thyroid-stimulating hormone (TSH), lipids, insulin and glucose) were measured in standard assays at the central laboratory.

The majority of patients had isolated GHD. Three (3%) were receiving hydrocortisone replacement treatment, and 9 (10%) were on T_4_ (evenly distributed between GHD severity groups). Molecular genetic studies to define the etiology of the GHD and MR scanning of the hypothalamic–pituitary axis were not required as part of the protocol.

Growth parameters were collected at baseline and on an annual basis. All were converted to SDS using the Sempé reference data (19).

During years 1, 2, and 3, subjects with severe GHD were treated with median GH doses of 34, 31, and 31 μg/kg per day, respectively, and subjects with mild GHD were treated with median doses of 35, 34, and 34 μg/kg per day respectively. There was no significant difference across the years of therapy in the dose received by children with mild GHD (rank sum *P*=0.98) or in the dose received by those with severe GHD (rank sum *P*=0.30).

We have previously demonstrated no confounding effects of country of origin or population stratification on the response to r-hGH after the first year of treatment (17).

### Genotyping assay

The genotyping of the two GHR exon 3 alleles (d3, exon 3 deletion; fl, full-length gene) was carried out using a gel-based triplex PCR technique published by Pantel *et al*. (20) All DNA samples were successfully genotyped and no deviation from the Hardy–Weinberg equilibrium (Fisher's exact test *P*>5%) was observed. No imbalance in genotype frequencies was found between subjects with mild and severe GHD (Fisher's exact test *P*=0.79). The numbers of d3/d3 homozygotes was small compared to d3/fl heterozygotes (Supplementary Table 1, see section on supplementary data given at the end of this article), therefore no further statistical analysis of genotype was performed. Subsequent to the determination of the GHR exon 3 alleles, genetic data were encoded as presence or absence of the d3 allele (d3 carriers, fl/fl).

### Statistical analysis

#### Time series analyses

Change over time in growth-related endpoints was tested using linear mixed-effect models (to model change in endpoints over 3 years of therapy). The interaction between GHRd3 polymorphism and GHD severity was modeled as a fixed effect and inter-individual variability as a random effect. These models are referred to as conditional models. Additive models, to test GHRd3 polymorphism and disease severity without interaction, were also used. For all models, gender was included as a covariate. The significance of each term (for all models) was tested using ANOVA type III. Levels in the variables were encoded as follows: GHD severity term (severe; mild); GHRd3 polymorphism (d3 carriers; fl/fl); and gender (female; male). In addition, an analysis of the impact of the GHRd3 polymorphism-GHD severity interaction on growth stratified by both country of origin of the patient and the study site was undertaken (data not shown).

#### Change in serum biomarker levels

Changes in biomarker levels after 1 month of therapy were assessed using linear models. The interaction between GHRd3 polymorphism and disease severity was tested with interaction models, while the effect from both terms (in the absence of interaction) was tested with additive models. All models included age at baseline and gender as covariates. The significance of each term was tested using ANOVA type III. *P* values were adjusted using the Benjamini–Hochberg correction. Levels in the variables were encoded as previously described.

#### Transcriptome analysis

In order to explore possible mechanisms related to the GHRd3-GHD severity association, GE profiling was performed at baseline on whole blood RNA extracted centrally by qLAB using the PAXgene 96 blood RNA kit (Qiagen). Reduction of globin messenger RNA was undertaken using the Ambion GLOBIN Clear Human kit (Life Technologies, Paisley, UK). Complementary RNA was generated using the Two-Cycle Eukaryotic Target Labelling kit (Affymetrix, Santa Clara, CA, USA) before hybridization to Affymetrix GeneChip Human Genome U133 Plus 2.0 Arrays.

Processing and normalization of GE data from each patient were performed using a Robust Multi-array Average background correction modified for probe sequence with quantile normalization and median polish (Partek Genomics Suite, version 6.3, St Louis, MO, USA). Confounding effects due to variations in cell populations and outliers were examined by cross validation using principal component analysis and iso-map multidimensional scaling (Qlucore Omics Explorer 2.2, Qlucore, Lund, Sweden).

The relationships between basal GE and GHD severity and basal GE and carriage of the GHRd3 polymorphism were assessed using rank regression and ANOVA as appropriate, adjusting for gender, ethnicity, age and baseline BMI as potential confounding factors (Qlucore Omics Explorer 2.2).

#### Causal network analysis of transcriptomic data

Causal network analysis (CNA) allows the identification and prioritisation of regulatory system elements within transcriptomic models. CNA was performed within Ingenuity Pathways Analysis (IPA, Redwood City, CA, USA) using the overlap of associated GE between GHD severity and carriage of the GHRd3 polymorphism.

CNA identifies upstream molecules up to three steps distant that control the expression of the genes in the dataset, and thus provides insight into information flow within the network (21). These relationships are derived from published literature and multiple database platforms curated in the Ingenuity Knowledge Base. A prediction of the activation state for each regulatory factor (master regulator), based on the direction of change, was calculated (*z*-score) using the GE patterns of the transcription factor and its downstream genes. An absolute *z*-score of ≥|1.4| and a false discovery rate (FDR) corrected *P* value <0.05 (Fisher's Exact test with Benjamini Hochberg correction) were used to compare the regulators identified in each of the four GE datasets (severe GHD:*fl/fl*GHR/severe GHD:GHRd3 carriage/mild GHD:* fl/fl*GHR/mild GHD:GHRd3 carriage) using hierarchical clustering (Euclidean metric).

#### Baseline GE and GH signaling

The predicted effect of basal GE in each of the four GHR genotype:GH status categories (severe GHD:*fl/fl*GHR/severe GHD:GHRd3 carriage/mild GHD:* fl/fl*GHR/mild GHD:GHRd3 carriage) on the activity of the genes for each of the molecules in the GH signalling pathways was determined using the Molecular Activity Prediction (MAP) tool in IPA.

#### Clinical Trial registration

Clinical trials registration numbers: NCT00256126 and NCT00699855. Classification: Growth hormone basic.

## Results

### Baseline characteristics

The study included 94 subjects with GHD (mild GHD, *n*=49; severe GHD, *n*=45; see Materials and methods for definition) with a 3-year follow-up. No significant differences were observed in baseline characteristics between the GHD groups (Table 1). Frequency of the GHR exon 3 deletion (d3 allele) was 36% for the mild GHD group and 36% for the severe GHD group. These frequencies are consistent with previous observations (2, 20). No significant differences in baseline characteristics were found between subjects with the fl/fl genotype and d3 carriers when assessed by GHD severity group (Supplementary Table 1).

### Growth response related to GHRd3 polymorphism carriage and GHD severity

**Figure 1:**
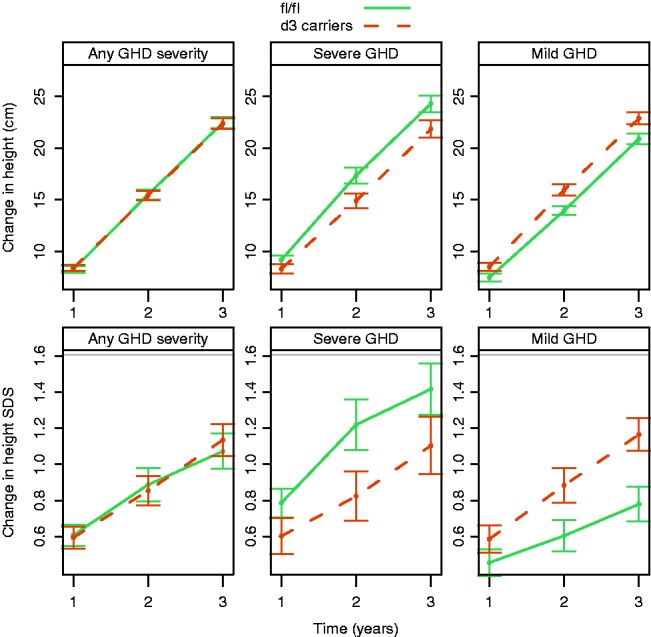
Change from baseline in height over time. Top panels show change in height (cm) and bottom panels show change in height SDS. Lines correspond to mean and error bars show the standard error of the mean. The interaction between GHRd3 polymorphism and GHD severity was significant for both endpoints (*P*=0.0018 and 0.010, respectively, for change in cm and SDS). GHD, growth hormone deficiency.

The impact of GHRd3 on change in height (cm) and change in height SDS during 3 years of GH treatment was tested (Fig. 1). When considering all subjects with GHD participating in this study (irrespective of their GHD severity), no significant association between GHRd3 polymorphism and growth response was found.

However, the relationship between the GHRd3 polymorphism and GH-dependent growth response variables was significantly influenced by the severity of GHD. Modeling change in height (cm) over time (using a linear mixed-effect framework) revealed a significant effect from the interaction between GHRd3 polymorphism and GHD severity (interaction *P*=0.0018; Fig. 1). In the group with severe GHD, d3 carriers had a significantly lower growth response compared with subjects having a full-length (fl/fl) GHR (differences in medians were −1.0, −2.6, and −3.3 cm for years 1, 2, and 3, respectively; Table 2). Conversely, in the group with mild GHD, d3 carriers had a higher growth response than subjects with fl/fl (differences in medians were +1.2,+2.0, and +2.7 cm for years 1, 2, and 3, respectively). An identical pattern was found for change in height SDS (interaction *P*=0.010; Fig. 1) and for height velocity SDS (interaction *P*=0.027). Age and r-hGH dose were excluded as potential confounding factors (data not shown). The country of origin and the study site also had no influence on the impact of the GHRd3 polymorphism-GHD severity interaction on growth responses (data not shown).

The significant interaction between GHRd3 polymorphism and GHD severity was not specific to the chosen statistical methodology, as all findings were confirmed with alternative approaches (non-parametric models). Furthermore, compared with a peak GH level cut-off of 4 μg/l to classify severity, the difference in height change between GHRd3 carriers and subjects with fl/fl with severe GHD was intensified when using a more stringent severity cut-off of 2 μg/l. Differences between GHRd3 vs fl/fl carriage at year 1 were −4.7 cm for height and −0.6 for height SDS.

The possible association of GHRd3 polymorphism and GHD severity with weight-related parameters, such as annualized BMI SDS and annualized weight SDS, was also tested. The GHRd3 polymorphism–GHD severity interaction was not significant for BMI (*P*=0.22) and only marginal for weight (*P*=0.059).

### Change in serum biomarkers after 1 month of therapy

**Figure 2:**
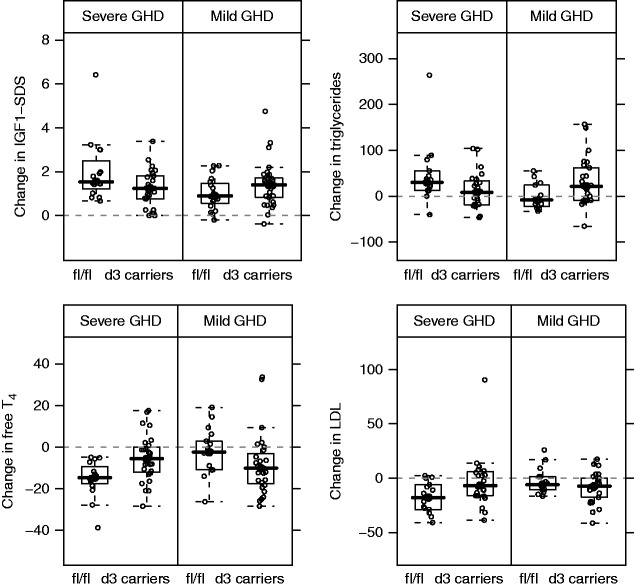
Change in serum biomarkers after 1 month of r-hGH therapy. Panels from top left to bottom right correspond to boxplots for change in IGF1 SDS, percentage change in fasting triglycerides (unit of measurement: mmol/l), percentage change in free T_4_ (pmol/l), and percentage change in fasting LDL-cholesterol (mmol/l). All interaction GHRd3 polymorphism–GHD severity terms were significantly associated with biomarker changes (FDR <5%). GHD, growth hormone deficiency; IGF1, insulin-like growth factor-I; T_4_, thyroxine.

The impact of 1 month of r-hGH treatment on the circulating concentrations of selected biomarkers (IGF1, IGFBP3, TSH, free T_4_, fasting glucose, insulin, cholesterol (HDL, LDL, and triglycerides) was investigated. Results from linear models are summarized in Table 3. As for growth response markers, a significant GHRd3 polymorphism–GHD severity interaction (FDR <5%) was found at 1 month for changes in IGF1 SDS, triglycerides, LDL-cholesterol, and free T_4_ levels (Fig. 2). A marginal interaction (FDR=13%) was found for change in fasting insulin levels. Regarding glucose–insulin metabolism, in subjects with severe GHD, fl/fl and d3 carriers did not differ in terms of changes in fasting insulin or homeostatic model assessment of insulin resistance (HOMA-IR). In contrast, in subjects with mild GHD, significant differences were found: d3 carriers had a+60% insulin increase (rank sum *P*=0.03) and a+66% HOMA-IR increase (rank sum *P*=0.07) compared with those who were fl/fl.

Overall, these biomarker patterns were consistent with those described for growth response markers; that is, subjects with severe GHD who were fl/fl (with the exception of insulin and HOMA-IR) and those with mild GHD who were d3 carriers had larger GH-dependent effects.

### Relationships between change in IGF1 SDS and change in height SDS

The impact of the GHRd3 polymorphism–GHD severity interaction on the relationship between height SDS and IGF1 SDS was investigated. When considering the whole GHD population, irrespective of their GHD severity or GHRd3 classification, change in height SDS after 1 year of therapy was associated with change in IGF1 SDS after 1 month of therapy (*P*=0.013). When the GHRd3 polymorphism–GHD severity interaction term was added into the model, both change in height SDS and the interaction term were significantly associated with change in IGF1 SDS (height SDS *P*=0.046 and interaction term *P*=0.023).

**Figure 3:**
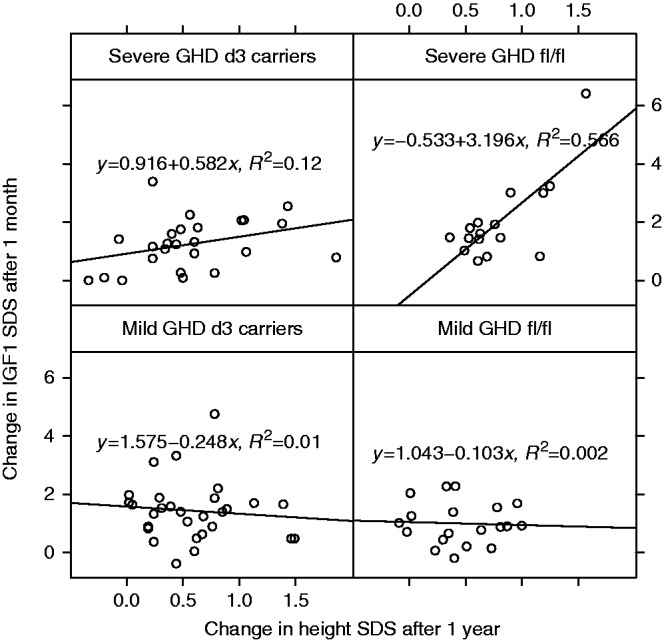
Correlation between change in IGF1 SDS and change in height SDS. Correlation is stratified by GHD severity–GHRd3 polymorphism groups. The line was fitted by a linear model, and the corresponding equation including *R*^2^ value is indicated in each panel. GHD, growth hormone deficiency; IGF1, insulin-like growth factor 1.

When results were analyzed separately for each GHD severity and GHRd3 category, a much stronger relationship between height SDS and IGF1 SDS in subjects with severe GHD than in those with mild GHD was observed (Fig. 3). The correlation between IGF1 and height change was strongest for subjects with severe GHD who were fl/fl (*r*=0.75 with 95% (CI: 0.41; 0.91), *P*=0.0008), and was weaker for those who were d3 carriers (*r*=0.35 with 95% CI (−0.05; 0.65), *P*=0.089*)*.The correlation for those with mild GHD, irrespective of GHRd3 status, was not significant (*P*>5%; Fig. 3).

### Relationship between baseline GE, GHD severity and GHRd3 carriage

The expression of 283 genes was significantly different between mild and severe GHD (rank regression, *P*<0.01). The expression of 457 genes was significantly different in those carrying a GHRd3 allele and those homozygous for *fl:fl* GHR (ANOVA, *P*<0.01) (Supplementary Table 2, see section on supplementary data given at the end of this article). The expression of nine genes overlapped between these two relationships (*P*<0.05, Supplementary Table 2), associated with cellular growth and proliferation pathways (*P*<1.0×10^−5^).

**Figure 4:**
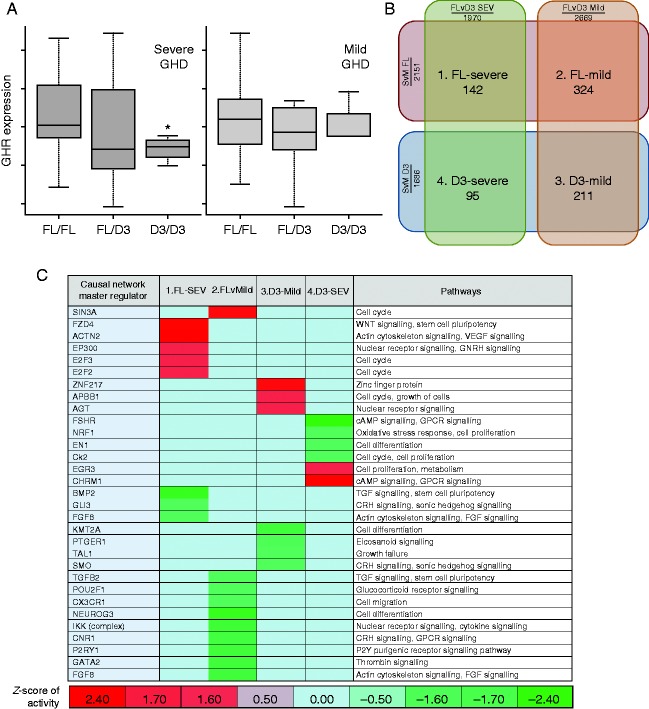
Gene expression associated with GHD severity and carriage of the GHRd3 variant. (A) Box and whisker plots of GHR expression by genotype (median and quartiles of Affymetrix probe-set 205498_at expression) (B) Diagram showing overlap of associated gene expression with severe compared to mild GHD and carriage of full length GHR compared to carriage of GHRd3 (*P*<0.05, numbers represent associated gene probe sets). (C) CNA was used to define master regulators associated with the regulation of the overlapping gene expression defined in (B) (modified *P* value <0.05 and *z*-score of activity >|1.4|). Data represented as a heat map with hierarchical clustering (Euclidean metric); biological pathways associated with master regulators are shown. The colour coding represents the predicted level of activity of the master regulator – deeper red represents increasing up-regulation (e.g. ACNT2) and deeper green represents increased down-regulation (e.g. BMP2).

The expression of the GHR gene within the transcriptome data set was determined in relation to GHD severity and GHR genotype. This analysis demonstrated lower GHR expression in D3 homozygotes compared to the *fl/fl* and *fl/d3* genotypes in severe GHD (*P*<0.05). This was not significant in mild GHD (Fig. 4A).

### Causal network modelling of GHRd3 carriage associated GE

The gene probe sets that overlapped between each pair: i) severe GHD:*fl/fl*GHR; ii) mild GHD:* fl/fl*GHR; iii) mild GHD:GHRd3 carriage; iv) severe GHD:GHRd3 carriage) were identified (Fig. 4B and Supplementary Table 2). CNA was then used to identify the regulators of the pathways represented in the overlapping GE profiles in the four groups (Fig. 4C). There was a predominance of pathways related to cell growth, cell cycle, cell differentiation and intracellular signalling. The regulators were ordered by hierarchical clustering (Fig. 4C), and this demonstrated that the ‘master’ regulator genes in each of the four groups were different, although the pathways being controlled often had a similar function. This indicated that the interaction between GHRd3 carriage and GHD severity has a distinct impact on GE. In the group with highest growth response (severe GHD:*fl/fl* GHR), *ACTN2* (a cytoskeleton protein) was the regulator with the highest activity and *BMP2* (an inducer of bone and cartilage formation) the lowest, while in the group with the poorest response (mild GHD:*fl/fl* GHR), *SIN3A* (a transcriptional regulator) was the regulator with the highest activity and *IKK* (an activator of NFκB) the lowest.

### Baseline GE and GH signaling

**Figure 5:**
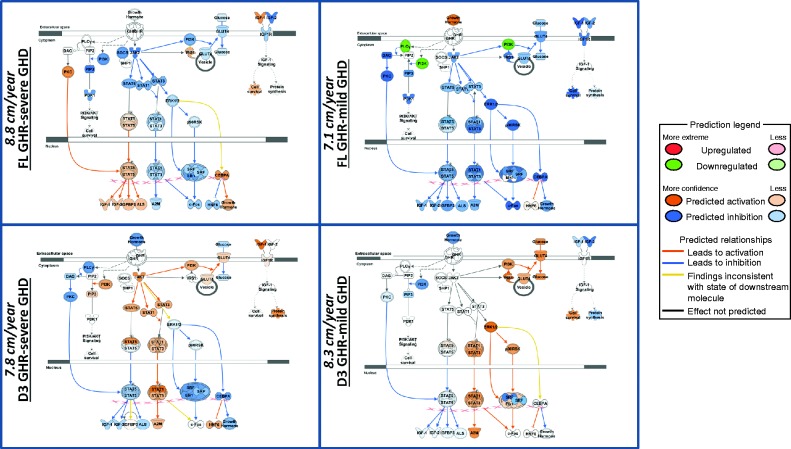
Predicted activity within the GH signal transduction pathways based on baseline gene expression. Each panel shows the signalling molecules in the GH pathways. The predicted level of expression (Orange, increased; Blue, decreased) of each of these molecules for each of the four GHRd3/GH status groups (FL-GHR-Severe GHD (38 genes), FL GHR-Mild GHD (64 genes), GHRd3-Mild GHD (48 genes), GHRd3-Severe GHD (20 genes)) is shown. The predicted levels of expression in the GH pathway are derived from the impact of the levels of baseline gene expression in each of the four states and their direct network interactions with the GH pathway. First year height velocity (cm/year) in each of the four states is shown in the left margin of each panel. The predicted action on the GH pathway molecules was determined using Molecular Activity Prediction (MAP) tool in IPA (see Legend below). The principal difference between GH deficient states for those with full-length GHR (top two panels) is that those with severe GHD are predicted to have an activated STAT5 pathway in the basal state. For carriage of GHRd3 (lower two panels), those with severe GHD are predicted to have inhibition in the ERK pathway in the basal state. When comparing between genotypes for both severe and mild GHD, those carrying GHRd3 have active STAT 1 and 3 pathways compared to inhibition for those with full-length GHR.

In order to assess whether the GE profiles associated with each of the four GHR genotype:GH status groups, the predicted effect on GH signalling molecules was quantified. This demonstrated significant differences in activity in the various GH pathways (Fig. 5). GH status had different impacts on signalling pathways dependent on GHR genotype (panel A vs B –*fl/fl* GHR: those with severe GHD are predicted to have an activated STAT5 pathway, and panel C vs D – GHRd3: those with severe GHD are predicted to have inhibition in the ERK pathway). When comparing between genotypes for both severe and mild GHD, those carrying GHRd3 have active Stat 1 and 3 pathways compared to inhibition for those with full-length GHR.

## Discussion

In the present study, the effect of r-hGH treatment on growth (over 3 years) and serum biomarker changes (at 1 month) in prepubertal children with GHD was investigated. It was found that treatment efficacy is influenced by GHRd3 polymorphism but modulated by GHD severity. The cohort was recruited from growth centers across the world, and purposely included children whose diagnosis of GHD was based on local criteria along with a peak GH level <10 μg/l in two stimulation tests. The cohort therefore included a wide range of GHD phenotypes from those with very severe GHD (the lowest peak GH level being 1 μg/l) to those with very mild GHD (the highest peak GH level being 9 μg/l). Some of the latter would be likely to retest as having normal GH status at the end of r-hGH treatment. We consider that having such a wide range of GH status provides strength to the study as i) it is reflection of current practice across many centers and ii) it provides an opportunity to test the full extent of the interaction between GHRd3 carriage and GH status. GH levels were assayed in the local centers using their preferred methodology. In the context of this study, it was not considered feasible to undertake a central assaying process for GH. If a single assay had been used, it is possible that some patients would be allocated to a different level of GHD severity. However, this would be most unlikely to be a systematic error and the observations in this study are consistent across all end-points assessed. For subjects with mild GHD, d3 carriers had a better growth response than those with the fl/fl genotype, in support of those studies that have previously reported the positive impact of GHRd3 carriage on growth during GH therapy (22). This positive impact had been described in a range of growth disorders including GHD, Turner syndrome, short SGA children and those with idiopathic short stature. The GHD studies had included children with a range of severity of GHD, but all were retrospective and an interaction between GHRd3 genotype and GH status was not examined in detail. The new finding in this study, and contrary to previous observations, is that subjects with severe GHD, who were homozygous for the full-length GHR (fl/fl) had a significantly better growth response than d3 carriers. The observed differences in r-hGH response were consistent over the 3 years of follow-up and are supported by similar findings for changes in IGF1 levels after 1 month of therapy. This observed interaction between GHD severity and GHRd3 polymorphisms is not explained by the administered r-hGH dose. In this study, a child was considered to have severe GHD if the peak GH response observed during their GH stimulation test was ≤4 μg/l. Importantly, our findings were reproduced when using a more stringent threshold (at a peak GH of ≤2 μg/l), which demonstrates that the observed GHRd3 polymorphism–GHD severity interaction is not dependent on a specific peak GH threshold. In fact, the difference in height change in subjects with severe GHD between GHRd3 carriers and those with the fl/fl genotype was intensified when a more stringent severity threshold was used. In addition, the effect of the interaction was not influenced by country of origin of the recruits, nor by the study site location. Notably the GHRd3 polymorphism and the severity of GHD had no major impact on any growth parameter at baseline (Table 1), indicating that the interaction between GHRd3 and GHD severity is only unveiled by r-hGH treatment.

The limitation of using local criteria for the diagnosis of GHD is that different provocation tests, GH assays and reference preparations will have been used. Therefore the peak GH values in some cases may not be directly comparable between subjects. As we have used cut-off values of 4 μg/l and 2 μg/l to distinguish between severe and mild GHD, then it is possible that some patients could be incorrectly allocated to one or other group. This may be the case, but it would be expected that this would be a random not a systematic error. In addition, we propose that the following features of the GHD group provide validity for using a cut-off level to define severity and indicate that bias has not been introduced: i) the expected negative relationship between growth response and peak GH value is seen in all 3 years of GH treatment; ii) consistent with the growth response, changes in IGF1 and IGFBP-3 SDS over the first month of GH treatment were negatively related to peak GH; iii) all the growth and metabolic endpoints showed consistent responses by severity of GHD and genotype (Tables 2 and 3); iv) using a more stringent cut-off of ≤2 μg/l to define severe GHD strengthened the observations; v) the GHR genotypes have been shown to be evenly distributed between the severity categories; vi) as indicated above, the study site had no effect on the analysis; and vii) analysis of the transcriptomic data supported the existence of an interaction between GHD severity and GHR genotype.

The significant interaction between GHR isoforms and GHD severity raises the possibility that genotype and GH level could influence the total number of GHRs expressed on a cell and/or efficiency of GH signaling and/or alter the activity in cellular pathways related to GH responsiveness. To explore the latter possibility, we used baseline GE profiling from whole blood to assess whether GHRd3 carriage and GH status could alter GE. Transcriptomic data from whole blood has been previously shown to be an effective model to study GH action (23) and human growth (24). First we examined the relationship between *GHR* genotype and GHD severity (Fig. 4A), and showed that *GHR* mRNA levels were significantly lower in *d3* homozygotes in those with severe GHD. Serum GH binding protein (GHBP) is derived from the proteolysis of GHR and, consistent with our observation in severe GHD, healthy *d3/d3* carriers present lower GHBP levels compared with *fl/fl* and *d3/fl* genotypes (25, 26). This is, however, unlikely to be the sole explanation for the GHR polymorphism/GH severity interaction.

Analysis of all the GE data, based on profiling in the baseline state, indicated that the prevailing level of GH secretion and the GHRd3 genotype did indeed have an impact on the genes being transcribed. The fact that differences in GE related to growth pathways have been demonstrated in the baseline GE shows that there are fundamental genomic differences between patients prior to treatment. We used causal network analysis to identify the ‘master’ regulators of this transcription and showed that these regulators differed between the four GHRd3 carriage:GHD severity groups (Fig. 4). This indicates that these ‘master’ genetic regulators are potential biomarkers of responsiveness to GH treatment in GHD and contribute new knowledge to the understanding of genetic mechanisms underlying responsiveness.

We also used the baseline GE profiles to identify genes in those profiles that interact with GH signaling molecules (Fig. 5). A predicted level of activity in each of these signaling molecules was identified. This demonstrated that GH status within each genotype and genotype within each GH status group had different impacts on the status of the GH signaling pathways. This provides direct *in-silico* evidence of how an interaction between genotype and GH status may translate into differences in growth.

The results also show that changes in IGF1, triglycerides, LDL-cholesterol, and free T_4_ after 1 month of r-hGH therapy can be explained by the interaction between GHRd3 and GHD severity (Table 3). Subjects who responded best to therapy (i.e. severe GHD–fl/fl and mild GHD–d3 carriers) exhibited greater increases in both IGF1 and triglyceride levels, and a greater decrease in free T_4_ levels. These observations are consistent with previous findings where a GH-induced decrease in free T_4_ levels (27, 28) was correlated with higher triglyceride levels (29, 30, 31). Subjects with the fl/fl genotype and severe GHD had a significantly greater decrease in LDL-cholesterol levels than d3 carriers with severe GHD, whereas there was no significant difference between fl/fl and d3 subjects with mild GHD.

The observed relationship between change in height and change in IGF1 further emphasizes the importance of the GHRd3 polymorphism–GHD severity interaction. Subjects with the fl/fl genotype and severe GHD showed a very strong correlation between growth response and IGF1 changes. In contrast, no correlation was observed for fl/fl subjects with mild GHD. This is intriguing and might suggest that growth response in these latter subjects is influenced by parameters other than IGF1, a proposal that is supported by the transcriptomic data. These data suggest that the GHRd3 polymorphism, shown to modulate responsiveness to GH using *in vitro* cellular models (2), may be associated with activation of different GH-signaling pathways, dependent on the level of endogenous GH present in the circulation. This also indicates that the response to exogenous GH is driven by multiple factors including genetic status as well as GHD severity.

In conclusion, this study shows that the interaction between GHD severity and GHRd3 polymorphism has a significant impact on responses to r-hGH. The controversy in the literature on the impact of the GHRd3 polymorphism is likely to be due to the complexity of this interaction and to the fact that statistical models previously used to test for the impact of GHRd3 on growth did not fully consider GHD severity. The fact that both growth and the metabolic effects of GH are modulated in the same way by the GHRd3 polymorphism–GHD severity interaction gives added confidence to our observations. In addition, we have demonstrated that baseline GE profiles are influenced by GHRd3 carriage and GH status. We suggest that our methodology provides guidance for the analytical design of subsequent trials and that GHD severity–GHRd3 stratification should be considered in future pharmacogenomic studies.

## Supplementary data

This is linked to the online version of the paper at http://dx.doi.org/10.1530/EJE-15-0474.

## Author contribution statement

A Valsesia performed the statistical analysis and generated the first draft of the manuscript supported by J Wojcik and B Destenaves. A Stevens performed the gene expression analysis and contributed to the writing of the manuscript. V A Peterkova, A Belgorosky, M Maghnie, F Antoniazzi, E Koledova and P Farmer contributed to analysis, writing and revision of the manuscript. This work was led by P Chatelain and P Clayton who oversaw the development of the analyses and manuscript.

## Figures and Tables

**Table 1:** Clinical characteristics at baseline for the GHD groups.

**Variable**^a^	**Any GHD**	**Severe GHD**	**Mild GHD**	***P* value**
*n*	94	45	49	–
Female gender (*n*)	31	14	17	–
Age (years)	9.0 (0.3)	8.9 (0.4)	9.2 (0.4)	0.6801
Height SDS	−2.3 (0.1)	−2.5 (0.2)	−2.2 (0.1)	0.2844
Weight SDS	−1.4 (0.1)	−1.2 (0.2)	−1.5 (0.1)	0.3719
Bone age (years)	6.8 (0.3)	6.7 (0.5)	7.0 (0.5)	0.6374
Distance to target height SDS	−1.4 (0.2)	−1.4 (0.3)	−1.3 (0.2)	0.7593
IGF1 SDS	−1.8 (0.1)^b^	−1.8 (0.2)^c^	−1.7 (0.2)^d^	0.5278
IGFBP-3 SDS	−0.3 (0.1)^b^	−0.4 (0.2)^c^	−0.2 (0.1)^d^	0.3455
Glucose (mmol/l)	4.8 (0.1)	4.7 (0.1)	4.8 (0.1)	0.4430
HOMA-IR	1.1 (0.1)	1.3 (0.7)	1.0 (0.1)	0.0766
Insulin (pmol/l)	38.2 (3.0)	43.3 (4.4)	32.7 (4.0)	0.0785
Total cholesterol (mmol/l)	4.8 (0.1)	4.7 (0.1)	4.9 (0.1)	0.2700
HDL-cholesterol (mmol/l)	1.7 (0.0)	1.6 (0.1)	1.8 (0.1)	0.0556
LDL-cholesterol (mmol/l)	2.7 (0.1)	2.7 (0.1)	2.8 (0.1)	0.6072
Triglycerides (mmol/l)	0.8 (0.0)	0.9 (0.1)	0.7 (0.1)	0.1679
Free T_4_ (pmol/l)	15.1 (0.2)	15.0 (0.3)	15.3 (0.3)	0.5174
TSH (mIU/l)	2.5 (0.2)	2.5 (0.2)	2.6 (0.3)	0.8378

HOMA-IR, homeostatic model assessment insulin resistance; IGFBP-3, insulin-like growth factor binding protein-3; IGF1, insulin-like growth factor 1; T_4_, thyroxine; TSH, thyroid-stimulating hormone.

a For continuous variables, the means (standard error of the mean) are shown for all GHD subjects (irrespective of GHD severity) and for GHD severity groups (severe: peak GH ≤4 μg/l, mild: peak GH >4 to <10 μg/l). The indicated *P* values were obtained from a Student's t test between mild and severe GHD. All baseline blood samples were taken under fasted conditions.

b *n*=92.

c *n*=43.

d *n*=49.

**Table 2:** Change in height from baseline: median and 95% bootstrap CI estimates for each year of r-hGH therapy.

**Endpoint**	**Severity**	**Year**	**fl/fl (median and 95% CI)**	**d3 carriers (median and 95% CI)**	**Difference (d3 carriers – fl/fl) **
Change in height (cm)	Mild GHD	1	7.1 (6.6; 8.2)	8.3 (7.4; 8.6)	1.2
		2	14.1 (13.2; 15.0)	16.1 (14.3; 16.5)	2.0
		3	21.0 (19.7; 21.4)	23.7 (21.5; 24.0)	2.7
	Severe GHD	1	8.8 (8.0; 10.2)	7.8 (7.0; 9.3)	−1.0
		2	16.5 (14.5; 19.2)	13.9 (13.0; 15.9)	−2.6
		3	24.1 (21.7; 26.4)	20.8 (19.6; 23.6)	−3.3
Change in height SDS	Mild GHD	1	0.4 (0.3; 0.6)	0.5 (0.3; 0.7)	0.1
		2	0.6 (0.3; 0.8)	0.8 (0.5; 1.0)	0.2
		3	0.8 (0.5; 1.0)	1.0 (0.9; 1.2)	0.2
	Severe GHD	1	0.6 (0.6; 0.8)	0.5 (0.4; 0.7)	−0.1
		2	1.2 (0.8; 1.2)	0.7 (0.4; 0.9)	−0.5
		3	1.4 (1.1; 1.6)	1.1 (0.6; 1.3)	−0.3

GHD, growth hormone deficiency; r-hGH, recombinant human growth hormone.

**Table 3:** Change or percentage change in serum biomarker levels after 1 month of r-hGH therapy.

**Biomarker**^a^	**FDR**	**Mild GHD (difference, d3 carriers – fl/fl)**^b^	**Severe GHD (difference, d3 carriers – fl/fl)**^b^
Triglycerides (mmol/l)^c^	0.0119	29.4	−21.0
Free T_4_ (pmol/l)^c^	0.0119	−7.7	9.0
LDL-cholesterol (mmol/l)^c^	0.0138	−1.1	11.1
IGF1 SDS	0.0138	0.5	−0.3
Total cholesterol (mmol/l)^c^	0.0564	1.8	6.6
Insulin (pmol/l)^c^	0.1354	60.0	−35.9
IGFBP-3 SDS	0.2591	0.1	0.0
HDL-cholesterol (mmol/l)^c^	0.2591	−3.8	7.9
Glucose (mmol/l)^c^	0.2591	4.4	−8.1
HOMA-IR^c^	0.2591	66.3	−28.3
Change in IGF1 SDS/IGFBP-3 SDS	0.3560	−0.3	−0.3
TSH (mIU/l)^c^	0.3560	−6.5	1.2

FDR, false discovery rate; HOMA-IR, homeostatic model assessment of insulin resistance; IGFBP-3, insulin-like growth factor binding protein-3; IGF1, insulin-like growth factor 1; T_4_, thyroxine; TSH, thyroid-stimulating hormone.

a Parameters are ranked by FDR, which corresponds to the interaction between GHRd3 polymorphism and GHD severity *P* value from a linear model adjusting for age at baseline and gender.

b Differences between the median value for subjects with the fl/fl genotype and the median value for d3 carriers are given for mild and severe GHD separately.

c Percentage change. Units of measurement are given in brackets.
